# Mixed Reality Ultrasound-Guided Mini-ECIRS with Apple Vision Pro™ - First Case Report

**DOI:** 10.1590/S1677-5538.IBJU.2024.0610

**Published:** 2025-01-03

**Authors:** Roberto Montoro, Fabio C. Vicentini, Ricardo T.S. Ugino, Alexandre Danilovic, Giovanni S. Marchini, Fabio C.M. Torricelli, Carlos A. Batagello, Anderson B. Pellanda, Alexandre Silva, William C. Nahas, Eduardo Mazzucchi

**Affiliations:** 1 Hospital Ana Costa Divisão de Urologia Santos SP Brasil Divisão de Urologia, Hospital Ana Costa, Santos, SP, Brasil; 2 Hospital Israelita Albert Einstein Divisão de Urologia São Paulo SP Brasil Divisão de Urologia, Hospital Israelita Albert Einstein, São Paulo, SP, Brasil;; 3 Hospital das Clínicas Universidade de São Paulo Divisão de Urologia São Paulo SP Brasil Divisão de Urologia, Hospital das Clínicas Universidade de São Paulo – USP, São Paulo, SP, Brasil; 4 Universidade Federal do Paraná Divisão de Urologia Curitiba PR Brasil Divisão de Urologia da Universidade Federal do Paraná - UFPR, Curitiba, PR, Brasil

## Abstract

**Introduction:**

Some endourological surgeries require multiple screens to perform combined procedures, which can present ergonomic challenges (1, 2). Apple Vision Pro (AVP) is a spatial computing device developed by Apple that incorporates virtual reality (VR) for life-like simulations, realistic medical scenarios, interactive anatomical models, and augmented reality (AR) technologies (3). In health care, VR is used for pain management, physical therapy, psychological therapy, and surgical simulations, providing a controlled and safe environment for both patients and healthcare professionals (4).

**Objective:**

To demonstrate the step-by-step technique of the Mini-Endoscopic Combined Intra-Renal Surgery (Mini-ECIRS) procedure guided by ultrasound and using mixed reality technology with the Apple Vision Pro (multiscreen and 3D reconstruction). To the best of our knowledge, this is the first report of this procedure being performed with AVP assistance.

**Patient and Methods:**

We present the case of a 40-year-old female with a history of right lumbar pain for one year. A CT scan revealed a proximal ureteral stone (20mm) and a lower pole stone (14mm) on the right side, with a Guys's Score grade 2 4. In this case, we opted for Ultrasound-Guided Mini-ECIRS (5, 6). This choice allowed for precise puncture and dilation, ensuring effective treatment and minimal invasiveness, assisted by the Apple Vision Pro. This device is equipped with eight external cameras that capture the real world at a resolution of 4K, enhancing the surgeon's experience with unparalleled efficiency and ease of mixed reality. This advanced imaging allows for precise visualization and integration of digital elements into the physical environment, significantly improving the accuracy and effectiveness of surgical procedures. During this procedure, the multitude of equipment in the operating room often obstructs the view of the physical monitors, including ultrasound. However, this technology addresses these challenges by offering enhanced ergonomics, efficiency, and safety to the surgeon. By providing seamless integration of digital overlays and real-world visuals, it ensures that crucial information is always within the surgeon's line of sight, thereby improving operational precision and overall outcomes. The surgeon had no previous contact with the AVP and was assisted by an AVP expert urologist throughout the procedure.

**Results:**

The procedure was performed in the Barts flank-free position. Initially, ureterolithotomy was performed using holmium laser. After the dusting phase, an ultrasound-guided renal puncture was performed using a virtual screen, providing enhanced comfort and ergonomics for the surgeon. Throughout the procedure, the surgeon had simultaneous access to both screens (nephroscope and flexible ureteroscope), facilitating efficient location of any residual stones. The AVP functioned effectively, displaying multiple screens within its own interface, improving ergonomics during surgery and maintaining safety throughout the procedure. The surgery was performed uneventfully in 2 hours, and the patient was rendered stone-free on CT and was discharged on the first postoperative day.

**Conclusion:**

Apple Vision Pro provides multiscreen and 3D reconstruction capabilities, ensuring a comfortable, safe, and easily replicable procedure. Its advanced technology may be particularly beneficial for surgeries, such as Mini-ECIRS, which require simultaneous screens.

Available at: http://www.intbrazjurol.com.br/video-section/20240610_Montoro_et_al
